# The Continuum of Severity of Functional Impairment Due to Indoor Air Symptoms: Prevalence and Determinants

**DOI:** 10.1097/JOM.0000000000002884

**Published:** 2023-05-23

**Authors:** Einar Eidstø, Sanna Selinheimo, Jussi Lampi, Anniina Salmela, Juha Pekkanen

**Affiliations:** From the Department of Public Health, Faculty of Medicine, University of Helsinki, Helsinki, Finland (E.E., S.S., J.P.); Finnish Institute of Occupational Health, Helsinki, Finland (S.S.); and Department of Health Security, Environmental Health Unit, Finnish Institute for Health and Welfare, Kuopio, Finland (J.L., A.S., J.P.).

**Keywords:** indoor air, symptoms, functional impairment, disability, population-based survey, epidemiology, comorbid diseases, socioeconomic factors, idiopathic environmental intolerance

## Abstract

**Objective:**

The aim of the study is to study functional impairment due to indoor air–related symptoms and associated characteristics.

**Methods:**

A questionnaire survey of a random sample of Finnish people aged 25 to 64 years. Analyses were done using multivariate multinomial logistic regression.

**Results:**

A total of 23.1% reported indoor air–related symptoms, 1.8% severe functional impairment due to symptoms, 5.3% moderate, 11.1% mild, and 4.9% reported no impairment. Those with severe functional impairment showed the strongest associations with comorbid diseases, for example, asthma and irritable bowel syndrome, perceived sensitivities to several environmental factors, like chemicals, and likelihood of having symptoms in multiple organs, while those with no or little functional impairment showed weak or even inverse associations. Similar results emerged with severity of indoor air–related symptoms.

**Conclusions:**

Individuals with indoor air–related symptoms are a very heterogeneous group. This should be better considered in future research and clinical practice.

## Abstract 2

Individuals with indoor air–related symptoms are a heterogeneous group. Those with more functional impairment due to symptoms have more somatic, psychiatric, and functional comorbidities. Multiple health problems may increase the risk for higher functional impairment and prevent individuals from gaining from interventions. This should be better considered in future research and clinical practice.

**LEARNING OUTCOMES**

- To be able to describe the prevalence of different levels of functional impairment due to indoor air–related symptoms among the adult population and associated factors.
- To understand the importance of assessing the differences in functional impairment due to indoor air–related symptoms.
- To remember to consider in their own clinical practice that people with more severe functional impairment due to indoor air–related symptoms also have more somatic, psychiatric, and functional comorbidities, which may prevent them from gaining from care and support.

Indoor air or building-related symptoms are common in nonindustrial environments.^1–5^ Symptoms range from organ specific, such as mucous-membrane irritation and dry skin to general, nonspecific symptoms of fatigue and headaches.^6^ Risk factors for indoor air–related symptoms include not only indoor air pollution, such as molds, chemicals, and poor ventilation, but also individual factors, such as sex, concurrent diseases, personality traits, and psychosocial factors at work.^3,7–10^ In addition to increasing symptoms, mold and dampness exposure has been shown to be associated with onset of new asthma also in adults.^11,12^

A few clinical studies have shown that some of the patients have difficult functional impairment due to persistent symptoms, meaning severe restrictions to personal, occupational, and social life. These persistent symptoms have been shown to be associated with limitations of daily activities due to, for example, avoidance of buildings that the individual perceives to trigger symptoms. In addition, adverse effects on work ability seem to be common among these individuals. In the most severe cases, disability to work persists despite environmental repairs, improvements in indoor air quality, or avoidance of the building in question.^13–18^ These persistent indoor air–related symptoms have been shown to affect quality of life even more than common diseases such as asthma or depression.^19^ However, the great majority of studies on indoor air–related symptoms do not consider the severity of symptoms or associated levels of functional impairment, but typically only symptom frequency.^2,20–22^

While symptoms and symptom severity are important for diagnosis, assessing functional capacity is also important in clinical practice. In addition to a biomedical model that focuses on the underlying disease, functional capacity considers the individual’s psychological, social, and physical abilities to perform in daily life.^23^ Hence, this study focuses on levels of functional impairment. Assessing the level of functional impairment helps identify, design, and target the support according to the individual’s needs, such as job accommodations, sick leave, or other social support and rehabilitation. In research, studying the levels of functional impairment helps determine more accurately the burden of different symptoms and diseases to society^24^ and thereby helps guide resource allocation in health care and public health policy. In addition, estimates of the prevalence of symptoms are not comparable between studies without considering the severity of symptoms or of functional impairment. One population-based study has been done among pregnant women on level of functional impairment due to environmental intolerance.^25^ However, to our knowledge, there are no previous population-based studies that would have explored the full range of the variability in the level of functional impairment, or of symptom severity, due to indoor air–related symptoms.

Therefore, the present study determined for the first time the prevalence of different levels of functional impairment due to indoor air–related symptoms in a representative, population-based sample. Furthermore, this study explored the associations of sociodemographic, clinical, and other characteristics with levels of functional impairment. Analyses were repeated also for severity of indoor air–related symptoms.

## METHODS

### Study Population

The National Survey on Indoor Air was conducted jointly by the Finnish Institute for Health and Welfare and the University of Helsinki from November 2018 to March 2019. A postal questionnaire was sent to a random sample of 4997 Finnish speakers (25–64 years old) in Finland, excluding Åland. A total of 1797 subjects (36%) responded either to the postal or the electronic questionnaire.

The study was approved by an institutional review board of the Finnish Institute for Health and Welfare.

### Health Outcomes and Variable Coding

Respondents were asked, “Have you ever gotten symptoms from indoor air at home?” The question had three options (no symptoms; yes, during the past 12 months; yes, over 12 months ago). The same question was then asked about indoor air symptoms at work. Those who reported symptoms during the past 12 months either at home or at work were considered symptomatic and those who reported no symptoms or had had symptoms only over 12 months ago were considered nonsymptomatic. Those with missing answers to these questions were classified as missing and excluded from further analyses.

Those individuals who reported symptoms were further asked “Have these symptoms made it difficult to work, manage your domestic responsibilities, or get along with other people during the past 12 months?” There were five response options (not at all, quite a little, moderately, quite a lot, a lot). For the analysis, the last two categories were combined, as there were only eight respondents in the last category, to form the following five groups: no symptoms, symptoms with no functional impairment, mild functional impairment, moderate functional impairment, and severe functional impairment.

In addition to functional impairment, respondents were asked about the self-perceived severity of indoor air–related symptoms and avoidance behavior (see Methods, Supplemental Digital Content 1, http://links.lww.com/JOM/B345). Self-perceived severity of symptoms was asked on a 4-point scale (mild, moderate, severe, very severe symptoms). Because of low numbers, the last two categories were combined to form the following four groups: no symptoms, mild symptoms, moderate symptoms, and severe symptoms. Participants were also asked about the measures to avoid indoor air symptoms they had taken during the past 12 months (Table 4, Supplemental Digital Content 1, http://links.lww.com/JOM/B345).

**TABLE 1:** Associations of Severity of Functional Impairment Due to Indoor Air-Related Symptoms With Symptom Severity, Avoidance Behavior, Physician Visits, and Sick Leave Due to Indoor Air–Related Symptoms in the Past 12 Months

	No Symptoms (*n* = 1,345*), %	Symptoms, No Functional Impairment (*n* = 86), %	Symptoms, Mild Functional Impairment (*n* = 193), %	Symptoms, Moderate Functional Impairment (*n* = 91), %	Symptoms, Severe Functional Impairment (*n* = 31), %
Severity of indoor air–related symptoms					
Mild symptoms	0	87.4	63.3	21.5	9.4
Moderate symptoms	0	8	34.2	66.7	21.9
Severe symptoms	0	0	1.5	10.8	62.5
Avoidance behavior†					
No avoidance	100	37.2	19.5	8.6	3.2
Slight avoidance	0	48.8	56.4	40.9	9.7
Moderate avoidance	0	12.8	16.4	30.1	35.5
Much avoidance	0	1.2	7.7	20.4	51.6
Physician visit†	0.4	2.3	7.2	27.5	53.1
Sick leave†	0.1	0	3.6	19.4	53.1
Symptoms at home	0.1	32.2	22.6	32.6	43.8
Symptoms at work	1.4	76.7	90.8	89.2	78.1

*Because of missing data, numbers vary among those with no symptoms between 1,345 and 1,362, with no functional impairment between 86 and 87, with mild functional impairment between 193 and 196, with moderate functional impairment between 91 and 93, and with severe functional impairment between 31 and 32.

†Because of indoor air–related symptoms in the past 12 months.

The respondents were also asked “Which symptoms have you had due to indoor air and how often in the last 12 months?” They were given a predetermined list of 18 symptoms, and they had five response options from “never” to “nearly every day.” In the analysis, symptoms were dichotomized (at least once or twice a week vs less often). The 18 different symptoms asked were classified into five groups. The group of symptoms was coded to be present, if at least one of the symptoms in the group was present. The group “respiratory and eye symptoms” included five symptoms (nasal congestion, hoarse voice, shortness of breath, cough, eye symptoms), “skin symptoms” were asked with only one question, “joint symptoms” included joint symptoms and pricking of limbs, “general symptoms” included five symptoms (headache, vertigo, nausea, fatigue, brain fog), and “other symptoms” included five symptoms (heart palpitations, perspiration, urinary incontinence, diarrhea, fever).

Participants were asked to estimate their health on a five-point scale from “good” to “bad.” In the analysis, health was dichotomized (good or fairly good vs average, fairly bad, or bad). Similarly, the quality-of-life question had five options from “very bad” to “very good,” and it was dichotomized (very good or good *vs* average, bad, or very bad). Ability to work was estimated by the Work Ability Score.^26^ This one-item scale consists of a worker’s self-assessment of his/her current ability compared with the lifetime best. It ranges from 0 (“no ability to work”) to 10 (“ability to work at its best”). In this study, we dichotomized it into poor to moderate work ability (0–7 points) and good to excellent work ability (8–10 points). The prevalence of 11 common diseases such as asthma or coronary artery disease and nine functional disorders was assessed with the question “During the past 12 months, have you had any of these health conditions diagnosed or treated by a physician?” The respondent was coded as having an “other functional disorder,” if they reported having one or more of the following: chronic fatigue syndrome, fibromyalgia, chronic pain syndrome, odor sensitivity, multiple chemical sensitivity, noise sensitivity, or electromagnetic sensitivity.^27–29^ Self-perceived sensitivity to odors, chemicals, poor indoor air quality, and noise was assessed with the question “Do you think that you get symptoms more easily than other people when exposed to the following factors?” The question had the following four response options (no; yes, a little more easily; yes, much more easily; yes, very much more easily). The question was dichotomized (much or very much more easily vs no or little more easily). Participants were asked “Do you smoke?” They had seven options from “not at all” to “daily, over 15 cigarettes a day.” In the analysis, smoking was recategorized into those who never smoked and those who at least sometimes smoked.

### Demographic Characteristics and Variable Coding

Respondents were asked whether they were living in “owner-occupied housing,” “a rented apartment,” “a housing cooperative or similar,” or “somewhere else.” In the analysis, this was dichotomized (owner-occupied housing vs other). Financial situation was assessed with the question “Do you have enough money in view of your needs?” There were five options (not at all, a little, moderately adequate, almost fully adequate, fully adequate). In the analysis, financial situation was dichotomized (poor or very poor vs at least moderately adequate). The respondents were given four options to describe their place of residence (inner city, suburbs, countryside population center, dispersed settlement). Place of residence was dichotomized (urban area vs countryside). Form of housing had four options (single-family home, semidetached house or terraced house, apartment building, somewhere else). In the analysis, form of housing was dichotomized (single-family home vs other).

Because of data confidentially issues, age was available only in 5-year categories, and it was treated as a continuous variable in the analyses. Employment status was dichotomized into “employed or studying” and “others,” education into “academic degree” and “no academic degree,” and marital status into “married” and “not married.”

## STATISTICAL ANALYSIS

Multivariate models were run with multinomial logistic regression. The main dependent variable used was functional impairment due to indoor air–related symptoms, but most analyses were repeated also for severity of indoor air–related symptoms and avoidance behavior. Analyses were adjusted for age, sex, employed/studying, education, and marital status—though in the analyses of Table 2, only sex and age were used as confounders. The results are presented as odds ratios (ORs), including 95% confidence intervals (CIs). In addition, an omnibus test, a global test of the differences between all of the functional impairment groups, is reported. Sensitivity analyses were conducted by analyzing separately those who reported symptoms only at home or at work.

**TABLE 2:** Associations of Demographic Characteristics With Severity of Functional Impairment Due to Indoor Air–Related Symptoms During the Past 12 Months

	No Symptoms (*n* = 1,339*)	Symptoms, No Functional Impairment (*n* = 86)	Symptoms, Mild Functional Impairment (*n* = 192)	Symptoms, Moderate Functional Impairment (*n* = 91)	Symptoms, Severe Functional Impairment (*n* = 31)	Omnibus Test
Sex						
Female, %	53.5	57.5	64.8	83.9	62.5	
OR (95% CI)	1 (reference)	1.17 (0.76–1.82)	1.60 (1.17–2.18)	4.52 (2.57–7.93)	1.45 (0.70–2.98)	<0.001
Age						
25–45 yr,† %	38.5	50.6	48.5	49.5	53.1	
OR (95% CI)	1 (reference)	1.63 (1.06–2.52)	1.50 (1.11–2.03)	1.56 (1.02–2.38)	1.81 (0.90–3.65)	0.003
Marital status						
Married, %	52.1	50	54.9	38.7	46.9	
OR (95% CI)	1 (reference)	1.02 (0.66–1.60)	1.22 (0.90–1.66)	0.60 (0.39–0.94)	0.94 (0.46–1.93)	0.11
Education						
Academic degree, %	37.7	43.7	40.9	50.5	45.2	
OR (95% CI)	1 (reference)	1.10 (0.70–1.73)	0.97 (0.71–1.34)	1.41 (0.90–2.20)	1.05 (0.50–2.21)	0.65
Employment						
Employed, %	69.7	80.5	91.3	79.3	71.9	
OR (95% CI)	1 (reference)	1.64 (0.95–2.84)	4.32 (2.58–7.22)	1.64 (0.97–2.77)	1.00 (0.46–2.20)	<0.001
Owner-occupied housing, %	78.7	79.1	77.9	68.8	58.1	
OR (95% CI)	1 (reference)	1.23 (0.71–2.14)	1.09 (0.75–1.59)	0.62 (0.38–1.01)	0.45 (0.21–0.97)	0.76
Self-reported financial situation						
At least moderately adequate,‡ %	85.3	89.5	84.1	73.1	65.6	
OR (95% CI)	1 (reference)	1.48 (0.73–3.01)	0.91 (0.60–1.37)	0.45 (0.28–0.74)	0.33 (0.16–0.70)	0.001
Place of residence						
Urban area, %	64.5	70.9	65.8	67.7	67.7	
OR (95% CI)	1 (reference)	1.23 (0.76–2.00)	0.97 (0.70–1.34)	1.02 (0.65–1.62)	0.99 (0.46–2.15)	0.94
Form of housing						
Single-family home, %	49.4	41.9	51	41.9	54.8	
OR (95% CI)	1 (reference)	0.82 (0.53–1.29)	1.18 (0.87–1.61)	0.81 (0.53–1.26)	1.53 (0.73–3.18)	0.37
Smoking, %	20.4	23	21.1	13.2	19.4	
OR (95% CI)	1 (reference)	1.19 (0.71–1.99)	1.09 (0.75–1.58)	0.66 (0.35–1.24)	0.96 (0.39–2.38)	0.62

Odd ratio (95% CI): all odds ratios besides those for sex and age are adjusted for sex and age.

Omnibus test: a global test of the difference between the functional impairment groups.

Functional impairment: restrictions to work, domestic responsibilities, or social life.

*Because of missing data, numbers vary among those with no symptoms between 1,339 and 1,362, with no functional impairment between 86 and 87, with mild functional impairment between 192 and 196, with moderate functional impairment between 91 and 93, and with severe functional impairment between 31 and 32.

†Compared with the reference group 46 to 64 yr.

‡Moderately adequate, almost fully adequate, or fully adequate financial situation.

CI, confidence interval; OR, odds ratio.

Statistical analyses were conducted in IBM SPSS 28.0 for Windows (SPSS Illinois, Chicago, IL).

## RESULTS

**FIGURE 1:**
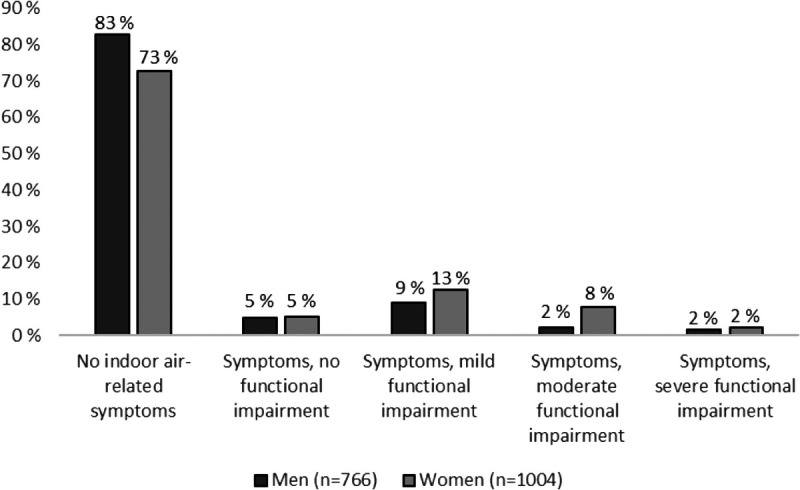
Prevalence of functional impairment (ie, difficulties with work, managing domestic responsibilities, or social life) due to indoor air–related symptoms in the past 12 months.

Of the respondents, 23.1% experienced indoor air–related symptoms, but only 18.2% reported functional impairment due to symptoms (Fig. 1). Most experienced mild functional impairment (11.1%), 5.3% moderate, and 1.8% severe functional impairment from indoor air–related symptoms. Of the respondents, 4.9% reported no functional impairment despite symptoms.

Severity of indoor air–related symptoms and amount of avoidance behavior due to these symptoms were strongly associated with severity of functional impairment (Table 1). Approximately two thirds of the subjects had the same degree of severity in both functional impairment and in symptom severity. Strong associations were also seen with visiting a physician and being on sick leave: while only 2.3% of those with symptoms, but no functional impairment, had visited a physician due to symptoms and none had been on sick leave, 53.1% of those with severe functional impairment had visited a physician and been on sick leave. While only a small portion of those with no functional impairment reported measures to avoid symptoms, a much larger portion of those with severe functional impairment did. Changing jobs and giving up a hobby had the largest relative increase with increasing functional impairment (Table 4, Supplemental Digital Content 1, http://links.lww.com/JOM/B345).

**TABLE 3:** Multivariate Adjusted Associations Between Doctor-Diagnosed Diseases and Severity of Functional Impairment* Due to Indoor Air–Related Symptoms During the Past 12 Months

	No Symptoms (*n* = 1,174†)	Symptoms, No Functional Impairment (*n* = 76)	Symptoms, Mild Functional Impairment (*n* = 162)	Symptoms, Moderate Functional Impairment (*n* = 79)	Symptoms, Severe Functional Impairment (*n* = 24)	Omnibus Test
Self-reported doctor-diagnosed or treated diseases during the past 12 mo						
Sleep apnea, %	5.4	10.4	6.1	8.8	16.7	
OR (95% CI)	1 (reference)	2.83 (1.27–6.35)	1.67 (0.81–3.44)	2.84 (1.19–6.77)	5.58 (1.72–18.13)	0.004
Asthma, %	7.3	9.2	10.8	25.6	25	
OR (95% CI)	1 (reference)	1.44 (0.64–3.25)	1.82 (1.05–3.16)	4.83 (2.71–8.61)	4.99 (1.88–13.20)	<0.001
Allergic rhinitis, %	15.6	31.2	30.4	42.7	57.7	
OR (95% CI)	1 (reference)	2.46 (1.47–4.10)	2.36 (1.62–3.43)	3.39 (2.08–5.52)	7.65 (3.36–17.41)	<0.001
Atopic eczema, %	9.7	10.5	16.4	22.8	25	
OR (95% CI)	1 (reference)	1.08 (0.50–2.31)	1.67 (1.04–2.68)	2.58 (1.45–4.60)	3.04 (1.17–7.90)	0.004
Depression, %	8.1	1.3	4.3	10.1	38.5	
OR (95% CI)	1 (reference)	0.15 (0.02–1.08)	0.62 (0.27–1.38)	1.20 (0.54–2.65)	7.64 (3.17–18.42)	<0.001
Irritable bowel syndrome, %	5.7	6.6	9.8	12.7	32	
OR (95% CI)	1 (reference)	1.49 (0.57–3.90)	2.35 (1.26–4.38)	2.79 (1.33–5.85)	10.22 (4.00–26.12)	<0.001
Sensitivity to poor indoor air quality, %	3.1	3.9	13.9	34.2	62.5	
OR (95% CI)	1 (reference)	1.47 (0.44–4.92)	5.70 (3.18–10.23)	17.23 (9.34–31.80)	64.77 (24.76–169.41)	<0.001
Other functional disorder,‡ %	7.6	6.6	11.8	29.6	37.5	
OR (95% CI)	1 (reference)	1.12 (0.43–2.91)	2.16 (1.23–3.78)	6.69 (3.71–12.07)	11.48 (4.45–29.65)	<0.001

Odds ratio (95% CI): OR and its 95% CI adjusted for age, sex, employed/studying, education, and marital status.

Omnibus test: a global test of the difference between the functional impairment groups.

*Functional impairment: restrictions to work, domestic responsibilities, or social life.

†Because of missing data, numbers vary among those with no symptoms between 1,174 and 1,200, with no functional impairment between 76 and 77, with mild functional impairment between 162 and 171, with moderate functional impairment between 79 and 82, and with severe functional impairment between 24 and 26.

‡Chronic fatigue syndrome, fibromyalgia, chronic pain syndrome, odor sensitivity, multiple chemical sensitivity, noise sensitivity, and electromagnetic sensitivity.

CI, confidence interval; OR, odds ratio.

Those with no functional impairment, mild functional impairment, or moderate functional impairment were significantly younger than the respondents without symptoms (Table 2). Especially those with moderate functional impairment were more often female and less often married. Particularly those with mild functional impairment were more often employed than the other groups. Respondents with moderate or severe functional impairment lived less often in owner-occupied housing and reported more often poorer financial situation. Smoking, place of residence, and form of housing had no significant associations with different levels of functional impairment.

Self-reported doctor-diagnosed allergic diseases, sensitivity to poor indoor air quality, irritable bowel syndrome, and other functional disorders were more common especially among those with severe functional impairment, but also among those with mild to moderate functional impairment (Table 3). Depression tended to be less common among those with no functional impairment, whereas it was significantly more common only among those with severe functional impairment.

**TABLE 4:** Multivariate Adjusted Associations of Health, Ability to Work, Quality of Life, and Environmental Sensitivities With Severity of Functional Impairment* Due to Indoor Air–Related Symptoms During the Past 12 Months

	No Symptoms (*n* = 1,335†)	Symptoms, No Functional Impairment (*n* = 84)	Symptoms, Mild Functional Impairment (*n* = 193)	Symptoms, Moderate Functional Impairment (*n* = 90)	Symptoms, Severe Functional Impairment (*n* = 32)	Omnibus Test
Self-reported health						
Good or fairly good, %	79.8	92	82.9	66.3	43.8	
OR (95% CI)	1 (reference)	2.25 (1.01–5.02)	0.84 (0.55–1.30)	0.32 (0.19–0.53)	0.11 (0.05–0.25)	<0.001
Self-reported ability to work						
Good or very good, %	75.9	82.8	83.5	66.3	40.6	
OR (95% CI)	1 (reference)	1.06 (0.57–1.95)	0.90 (0.59–1.39)	0.41 (0.25–0.69)	0.11 (0.05–0.25)	<0.001
Self-reported quality of life						
Good or very good, %	84	90.7	83.7	69.2	37.5	
OR (95% CI)	1 (reference)	1.53 (0.72–3.28)	0.66 (0.43–1.02)	0.31 (0.19–0.52)	0.07 (0.03–0.17)	<0.001
Self-perceived sensitivity to experience symptoms‡ from						
Odors, %	7.7	7.1	20.6	36.7	34.4	
OR (95% CI)	1 (reference)	1.05 (0.44–2.48)	3.85 (2.51–5.91)	6.51 (3.92–10.80)	7.24 (3.28–15.97)	<0.001
Chemicals, %	6.1	7.1	13.9	27.8	43.8	
OR (95% CI)	1 (reference)	1.48 (0.61–3.55)	3.56 (2.15–5.90)	6.63 (3.79–11.59)	15.43 (6.97–34.12)	<0.001
Poor indoor air quality, %	6.6	8	21.6	48.4	84.4	
OR (95% CI)	1 (reference)	1.29 (0.58–2.90)	3.99 (2.60–6.12)	11.68 (7.17–19.03)	78.95 (29.07–214.39)	<0.001
Noise, %	5.4	5.9	15.5	25.6	31.3	
OR (95% CI)	1 (reference)	1.18 (0.46–3.03)	3.52 (2.16–5.74)	5.55 (3.18–9.67)	8.33 (3.70–18.77)	<0.001

Odds ratio (95% CI): odds ratio and its 95% CI adjusted for age, sex, employed/studying, education, and marital status.

Omnibus test: a global test of the difference between the functional impairment groups.

*Functional impairment: restrictions to work, domestic responsibilities, or social life.

†Because of missing data, numbers vary among those with no symptoms between 1,335 and 1,353, with no functional impairment between 84 and 87, with mild functional impairment between 193 and 196, with moderate functional impairment between 90 and 92, and with severe functional impairment between 32.

‡Much or very much more easily than other people.

CI, confidence interval; OR, odds ratio.

Those with moderate and especially those with severe functional impairment reported poorer health, poorer ability to work, and lower quality of life (Table 4) than other respondents. Interestingly, those with no functional impairment despite symptoms had better self-reported health than respondents without symptoms. The prevalence of self-perceived sensitivity to environmental factors, such as odors, chemicals, poor indoor air quality, and noise, increased strongly with exacerbating severity of functional impairment when compared with those without symptoms.

Using severity of symptoms (Tables 1–3, Supplemental Digital Content 1, http://links.lww.com/JOM/B345) instead of functional impairment (Tables 2–4) due to indoor air–related symptoms yielded very similar associations, although severity of functional impairment showed in general slightly stronger associations than severity of symptoms. This was true especially for self-reported financial situation (Table 2), sleep apnea, depression, irritable bowel syndrome, and other functional disorders (Table 3), and self-reported health, ability to work and quality of life (Table 4), whereas asthma, atopic eczema (Table 3), and sensitivity to experience symptoms from odors, chemicals, or poor indoor air quality (Table 4) were somewhat more strongly associated with severity of symptoms. In addition, avoidance behavior produced mostly similar, but weaker associations (see Table 5, Supplemental Digital Content 1, http://links.lww.com/JOM/B345) than functional impartment or severity of symptoms. However, amount of avoidance behavior had no significant association with depression.

**FIGURE 2:**
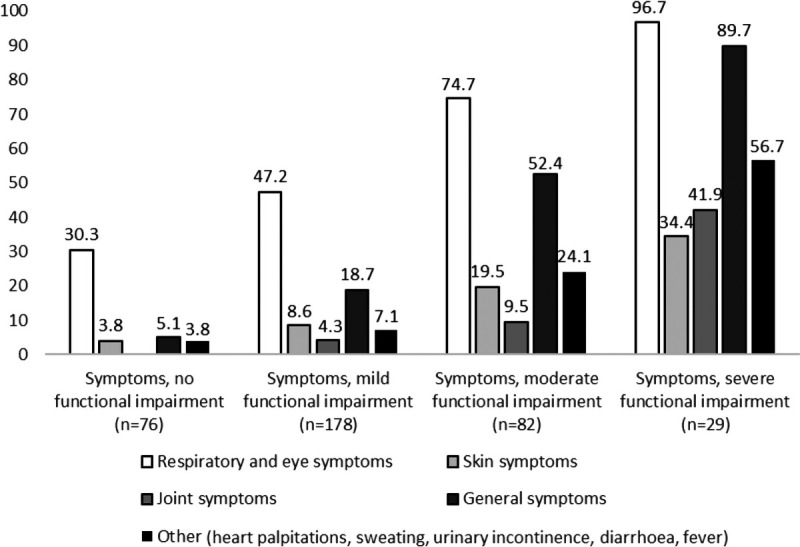
Proportion of self-reported symptoms (at least once or twice a week) by different levels of functional impairment.

Those with no or mild functional impairment reported mainly respiratory and eye symptoms, whereas among those with severe functional impairment, general symptoms were almost as common as respiratory and eye symptoms (Fig. 2). The prevalence of all groups of symptoms increased with increasing functional impairment. The symptoms with the strongest relative increases were joint symptoms, general symptoms, and other symptoms.

As sensitivity analyses, we also explored associations separately for those with indoor air–related symptoms at work and at home (data not shown). The small number of those with symptoms at home (*n* = 116), especially in the moderate (*n* = 30) and severe functional impairment (*n* = 14) groups, made the associations unstable. The main differences between those with symptoms at home compared with those with symptoms at work were that those with symptoms at home were less often employed, married, or had an adequate financial situation and had more often depression. This was true particularly among those with severe functional impairment. The associations for those with symptoms at work were very similar to those presented in Tables 2–4.

## DISCUSSION

The present population-based study shows that subjects reporting indoor air–related symptoms are a heterogeneous group. Two thirds of the individuals with indoor air–related symptoms report at most mild functional impairment, that is, difficulties with work, managing domestic responsibilities, or social life, due to indoor air–related symptoms. The prevalence of comorbid diseases, self-perceived sensitivities to environmental factors, symptoms in multiple organs, and to some extent poorer financial situation were more common among those with more severe functional impairment, whereas the associations were weaker or even inverse among those with no or mild functional impairment. Similar results were seen with severity of indoor air–related symptoms.

The present study is, to our knowledge, the first study exploring the levels of functional impairment and symptom severity due to indoor air–related symptoms in a representative population-based sample. Functional impairment among those with indoor air–related symptoms has earlier been explored in a few small-scale or qualitative^13,30–33^ and quantitative^14–18^ studies and have therefore focused on those with more severe symptoms. Two prospective studies^16,17^ have shown that a higher number of symptoms is associated with poorer work ability at follow-up.

Individuals with indoor air–related symptoms have been shown to have comorbidity with several diseases, both somatic and psychiatric.^3,20,34^ Our study is the first to show that the prevalence of comorbidities increases along with the level of functional impairment due to indoor air–related symptoms in the population. For example, while 10.8% of those with mild functional impairment had asthma, compared with 7.3% of individuals without symptoms, a quarter of those with severe functional impairment had asthma. The latter estimate is consistent with the estimate from a previous population-based study from Finland.^20^ Earlier studies based on data from occupationally active individuals have shown that those with indoor air–related symptoms have depression more often than nonsymptomatic individuals.^3,35^ We, however, found that depression was more common only among those with severe functional impairment, whereas the prevalence was decreased among those with no or mild functional impairment.

We observed that increasing severity of functional impairment due to indoor air–related symptoms was associated with an increased risk of reporting intolerance not only to indoor air but also to other environmental exposures. This finding is consistent with earlier findings from population-based studies in Finland^25^ and Sweden^36^ showing strong overlap between different idiopathic environmental intolerances, conditions that are characterized by symptoms from multiple-organ systems attributed to various environmental factors that are tolerated by most people.^37^ As observed before for environmental intolerance,^25^ also in the present study, the likelihood of having symptoms from multiple organs increased with increasing severity of functional impairment. We further observed a similar increased risk of having disorders that are labeled as functional somatic syndromes, like irritable bowel syndrome and chronic fatigue syndrome, with increasing severity of functional impairment.^27^ These findings are consistent with earlier suggestions that there is substantial overlap between the functional somatic syndromes or manifestations of persistent physical symptoms,^27^ which may result from shared predisposing and perpetuating factors and common underlying mechanisms. For example, controlled studies done among those with increased sensitivity to electromagnetic fields^38^ and multiple chemicals^39^ have shown that the symptoms among these individuals are not due to the suspected exposures, but knowledge or suspicion of being exposed. There is also extensive evidence that in addition to indoor air impurities and dampness and mold,^12,40–42^ indoor air–related symptoms are associated with other biopsychosocial factors as well, such as gender, stress, lack of social support, anxiety, and job demands,^2,3,7,9,40,43^ that may serve as perpetuating factors for functional impairment. In addition, we observed in the present study that the number of comorbidities was increased especially among those with severe functional impairment. Taken together, these observations suggest that factors other than indoor air impurities may play a larger etiological role among those with more severe functional impairment, which is also consistent with the clinical experience.

Those with lower socioeconomic status have been shown to have more diseases, lower self-perceived health, and higher mortality.^44^ However, no previous study has shown similar associations with indoor air–related symptoms. Our findings indicate that the more functional impairment due to indoor air symptoms one has, the more likely one is to report a worse financial situation. This may be due to inability to work because of symptoms. Indeed, self-reported ability to work was significantly lower among those with moderate and severe functional impairment (Table 4). Although the causality of the factors cannot be assessed, our results suggest that those with more functional impairment may be financially in a disadvantaged position compared with those with mild or no symptoms. As a similar trend was not seen for education, which is another commonly used indicator of socioeconomic status, more research is clearly needed on the strength and origins of the socioeconomic gradient in indoor air–related health and on the best ways to reduce this gradient.

In the present study, severity of symptoms was strongly related to severity of functional impairment due to these symptoms. In addition, almost all the observed results were very similar when using either severity of symptoms or functional impairment as the outcome. However, the two groups, while overlapping by approximately two thirds, were not the same and, in general, stronger associations were observed with functional impairment than with severity of symptoms. Compared with those with severe functional impairment, those with severe symptoms reported better health, better ability to work, or better quality of life. Possibly because of these reasons, fewer of those with severe symptoms reported being in a poor financial situation. In addition, severe functional impairment was clearly more strongly associated with depression. These differences support the importance of assessing the functional impairment of the patient in a clinical setting and in research, in addition to severity of symptoms.

One of the major strengths of this study is that it is the first population-based study describing different levels of functional impairment due to indoor air–related symptoms. The participation rate was only 36%. However, because no participation bias was detected for indoor air–related symptoms^45^ when compared with a previous study with a higher participation rate,^46^ the sample can be considered suitable for the present purpose. This study also had several limitations. The data were self-reported, and no clinical measurements were performed to assess the level of functional impairment or the presence of chronic diseases. We also did not have information on the respondents’ income. Another limitation of the study is its cross-sectional study design, which precludes the assessment of causality. In general, the direction of causality of the observed associations is difficult to assess. Indoor air–related symptoms may worsen the psychosocial burden, for example, increase stress, but psychosocial factors may also increase symptom reports. More longitudinal studies are therefore urgently needed.^47,48^

In conclusion, the present results show that the degree of functional impairment due to indoor air–related symptoms is a continuum in the population and that individuals with indoor air–related symptoms are a very heterogeneous group. When trying to understand this heterogeneity, other biological, psychological, and social factors^2,3,7,40^ need to be considered in addition to indoor air quality. The majority of those with symptoms have at most mild functional impairment and differ less from those with no symptoms. The more severe functional impairment one has, the more likely one is to have comorbidities, self-reported environmental sensitivities, symptoms in multiple organs, and possibly a poorer financial situation. Similar results were seen with severity of symptoms. Because of this heterogeneity, the determinants and etiologies of symptoms and functional impairments of differing severity likely differ and thus there is also a need for different types of treatments and support. Therefore, the heterogeneity of indoor air–related symptoms should be better considered in future studies on indoor air and health, as well as clinical practice.

## Supplementary Material

SUPPLEMENTARY MATERIAL

## References

[1] Bluyssen PM Roda C Mandin C, . Self-reported health and comfort in “modern” office buildings: first results from the European OFFICAIR study. *Indoor Air*. 2016;26:298–317. 25727348 10.1111/ina.12196

[2] Runeson-Broberg R Norback D. Sick building syndrome (SBS) and sick house syndrome (SHS) in relation to psychosocial stress at work in the Swedish workforce. *Int Arch Occup Environ Health*. 2013;86:915–922. 23143072 10.1007/s00420-012-0827-8

[3] Magnavita N. Work-related symptoms in indoor environments: a puzzling problem for the occupational physician. *Int Arch Occup Environ Health*. 2015;88:185–196. 24917077 10.1007/s00420-014-0952-7

[4] Selinheimo S Lampi J Pekkanen J. Parent's self-reported indoor environment-related symptoms and health worry increase symptom reports among their children at school-study in two independent populations. *Indoor Air*. 2021;31:1298–1307. 33955596 10.1111/ina.12836

[5] Park J Gilmour H. Medically unexplained physical symptoms (MUPS) Among adults in Canada: comorbidity, health care use and employment. *Health Rep*. 2017;28:3–8. 28295128

[6] Redlich CA Sparer J Cullen MR. Sick-building syndrome. *Lancet*. 1997;349:1013–1016. 9100639 10.1016/S0140-6736(96)07220-0

[7] Azuma K Ikeda K Kagi N Yanagi U Osawa H. Evaluating prevalence and risk factors of building-related symptoms among office workers: seasonal characteristics of symptoms and psychosocial and physical environmental factors. *Environ Health Prev Med*. 2017;22:38. 29165170 10.1186/s12199-017-0645-4 PMC5664800

[8] Norback D. An update on sick building syndrome. *Curr Opin Allergy Clin Immunol*. 2009;9:55–59. 19532093 10.1097/ACI.0b013e32831f8f08

[9] Bakke JV Moen BE Wieslander G Norback D. Gender and the physical and psychosocial work environments are related to indoor air symptoms. *J Occup Environ Med*. 2007;49:641–650. 17563607 10.1097/JOM.0b013e31806e5fa0

[10] Brasche S Bullinger M Morfeld M Gebhardt HJ Bischof W. Why do women suffer from sick building syndrome more often than men?—subjective higher sensitivity versus objective causes. *Indoor Air*. 2001;11:217–222. 11761596 10.1034/j.1600-0668.2001.110402.x

[11] Norback D Zock JP Plana E, . Mould and dampness in dwelling places, and onset of asthma: the population-based cohort ECRHS. *Occup Environ Med*. 2013;70:325–331. 23396522 10.1136/oemed-2012-100963

[12] Hurrass J Heinzow B Aurbach U, . Medical diagnostics for indoor mold exposure. *Int J Hyg Environ Health*. 2017;220(2 Pt B):305–328. 27986496 10.1016/j.ijheh.2016.11.012

[13] Vuokko A Karvala K Suojalehto H, . Clinical characteristics of disability in patients with indoor air–related environmental intolerance. *Saf Health Work*. 2019;10:362–369. 31497334 10.1016/j.shaw.2019.06.003 PMC6717934

[14] Al-Ahmad M Manno M Ng V Ribeiro M Liss GM Tarlo SM. Symptoms after mould exposure including *Stachybotrys chartarum*, and comparison with darkroom disease. *Allergy*. 2010;65:245–255. 19796210 10.1111/j.1398-9995.2009.02157.x

[15] Edvardsson B Bergdahl J Eriksson N Stenberg B. Coping and self-image in patients with symptoms attributed to indoor environment. *Arch Environ Occup Health*. 2013;68:145–152. 23566321 10.1080/19338244.2012.676102

[16] Edvardsson B Stenberg B Bergdahl J Eriksson N Linden G Widman L. Medical and social prognoses of non-specific building-related symptoms (sick building syndrome): a follow-up study of patients previously referred to hospital. *Int Arch Occup Environ Health*. 2008;81:805–812. 17924130 10.1007/s00420-007-0267-z

[17] Karvala K Nordman H Luukkonen R Uitti J. Asthma related to workplace dampness and impaired work ability. *Int Arch Occup Environ Health*. 2014;87:1–11. 23208737 10.1007/s00420-012-0830-0

[18] Karvala K Uitti J Luukkonen R Nordman H. Quality of life of patients with asthma related to damp and moldy work environments. *Scand J Work Environ Health*. 2013;39:96–105. 22407188 10.5271/sjweh.3289

[19] Selinheimo S Vuokko A Hublin C, . Health-related quality among life of employees with persistent nonspecific indoor-air–associated health complaints. *J Psychosom Res*. 2019;122:112–120. 30935665 10.1016/j.jpsychores.2019.03.181

[20] Karvala K Sainio M Palmquist E Claeson AS Nyback MH Nordin S. Building-related environmental intolerance and associated health in the general population. *Int J Environ Res Public Health*. 2018;15. 10.3390/ijerph15092047 PMC6163389 30235805

[21] Zhang X Sahlberg B Wieslander G Janson C Gislason T Norback D. Dampness and moulds in workplace buildings: associations with incidence and remission of sick building syndrome (SBS) and biomarkers of inflammation in a 10 year Follow-Up study. *Sci Total Environ*. 2012;430:75–81. 22634552 10.1016/j.scitotenv.2012.04.040

[22] Eriksson NM Stenberg BG. Baseline prevalence of symptoms related to indoor environment. *Scand J Public Health*. 2006;34:387–396. 16861189 10.1080/14034940500228281

[23] Cieza A Sabariego C Bickenbach J Chatterji S. Rethinking disability. *BMC Med*. 2018;16:14. 29370847 10.1186/s12916-017-1002-6 PMC5785824

[24] Vos T. In: Murray CJL Salomon JA Mathers CD Lopez AD, , eds. *“The Case Against Annual Profiles for the Valuation of Disability Weights”, in Summary Measures of Population Health: Concepts, Ethics, Measurement and Applications*. Geneva: World Health Organization; 2002.

[25] Vuokko A Karvala K Lampi J, . Environmental intolerance, symptoms and disability among fertile-aged women. *Int J Environ Res Public Health*. 2018;15:293. 29419757 10.3390/ijerph15020293 PMC5858362

[26] Gould R Ilmarinen J Järvisalo J Koskinen S. Dimensions of work ability. In: *Results From the Health 2000 Survey*. Helsinki, Finland: Finnish Institute of Occupational Health; 2008:25–34.

[27] Henningsen P Zipfel S Sattel H Creed F. Management of functional somatic syndromes and bodily distress. *Psychother Psychosom*. 2018;87:12–31. 29306954 10.1159/000484413

[28] Barsky AJ Borus JF. Functional somatic syndromes. *Ann Intern Med*. 1999;130:910–921. 10375340 10.7326/0003-4819-130-11-199906010-00016

[29] Dantoft TM Nordin S Andersson L Petersen MW Skovbjerg S Jorgensen T. Multiple chemical sensitivity described in the Danish general population: cohort characteristics and the importance of screening for functional somatic syndrome comorbidity—the DanFunD study. *PLoS One*. 2021;16:e0246461. 33626058 10.1371/journal.pone.0246461 PMC7904225

[30] Soderholm A Ohman A Stenberg B Nordin S. Experience of living with nonspecific building-related symptoms. *Scand J Psychol*. 2016;57:406–412. 27532686 10.1111/sjop.12319

[31] Finell E Seppala T Suoninen E. “It was not me that was sick, it was the building”: rhetorical identity management strategies in the context of observed or suspected indoor air problems in workplaces. *Qual Health Res*. 2018;28:1366–1377. 29441816 10.1177/1049732317751687

[32] Finell E Seppala T. Indoor air problems and experiences of injustice in the workplace: a quantitative and a qualitative study. *Indoor Air*. 2018;28:125–134. 28741743 10.1111/ina.12409

[33] Vuokko A Selinheimo S Sainio M, . Decreased work ability associated to indoor air problems—an intervention (RCT) to promote health behavior. *Neurotoxicology*. 2015;49:59–67. 26014487 10.1016/j.neuro.2015.04.010

[34] Claeson AS Andersson H Wikdahl F Nyback MH Nordin S. Comorbidity of airway inflammatory diseases in chemical and building-related intolerance. *J Occup Environ Med*. 2018;60:295–300. 29227362 10.1097/JOM.0000000000001249

[35] Kinman G Griffin M. Psychosocial factors and gender as predictors of symptoms associated with sick building syndrome. *Stress and Health*. 2008;24:165–171.

[36] Palmquist E Claeson AS Neely G Stenberg B Nordin S. Overlap in prevalence between various types of environmental intolerance. *Int J Hyg Environ Health*. 2014;217(4–5):427–434. 24029726 10.1016/j.ijheh.2013.08.005

[37] IPCS/WHO, Conclusions and recommendations of a workshop on multiple chemical sensitivities (MCS). *Regul Toxicol Pharmacol*. 1996;24:188–189.

[38] Rubin GJ Hillert L Nieto-Hernandez R van Rongen E Oftedal G. Do people With idiopathic environmental intolerance attributed to electromagnetic fields display physiological effects when exposed to electromagnetic fields? A systematic review of provocation studies. *Bioelectromagnetics*. 2011;32:593–609. 21769898 10.1002/bem.20690

[39] Das-Munshi J Rubin GJ Wessely S. Multiple chemical sensitivities: a systematic review of provocation studies. *J Allergy Clin Immunol*. 2006;118:1257–1264. 17137865 10.1016/j.jaci.2006.07.046

[40] Sakellaris I Saraga D Mandin C, . Association of subjective health symptoms with indoor air quality in European office buildings: the OFFICAIR project. *Indoor Air*. 2021;31:426–439. 32966653 10.1111/ina.12749

[41] Azuma K Ikeda K Kagi N Yanagi U Osawa H. Physicochemical risk factors for building-related symptoms in air-conditioned office buildings: ambient particles and combined exposure to indoor air pollutants. *Sci Total Environ*. 2018;616-617:1649–1655. 29070452 10.1016/j.scitotenv.2017.10.147

[42] Sun Y Hou J Cheng R Sheng Y Zhang X Sundell J. Indoor air quality, ventilation and their associations with sick building syndrome in Chinese homes. *Energ Buildings*. 2019;197:112–119.

[43] Marmot AF Eley J Stafford M Stansfeld SA Warwick E Marmot MG. Building health: an epidemiological study of “sick building syndrome” in the Whitehall II study. *Occup Environ Med*. 2006;63:283–289. 16556750 10.1136/oem.2005.022889 PMC2078095

[44] Mackenbach JP Stirbu I Roskam AJ, . Socioeconomic inequalities in health in 22 European countries. *N Engl J Med*. 2008;358:2468–2481. 18525043 10.1056/NEJMsa0707519

[45] Pekkanen J Jousilahti P Tiina L. Indoor air-associated symptoms (in Finnish). In: Koponen P Borodulin K Lundqvist A Sääksjärvi K Koskinen S, , eds. *Health, Functional Capacity and Welfare in Finland – FinHealth 2017 study*. Helsinki: National Institute for Health and Welfare; 2018:90–91.

[46] Borodulin K Sääksjärvi K, eds. *FinHealth 2017 Study – Methods*. Helsinki: Finnish Institute for Health and Welfare; 2019:132.

[47] Finell E Tolvanen A Pekkanen J Minkkinen J Stahl T Rimpela A. Psychosocial problems, indoor air–related symptoms, and perceived indoor air quality among students in schools without indoor air problems: a longitudinal study. *Int J Environ Res Public Health*. 2018;15:1497. 30012972 10.3390/ijerph15071497 PMC6069432

[48] Palmquist E. Stenberg B. Neely G., and Nordin S.. Environmental intolerance and mental ill-health: which comes first? 2017.

