# Supplementary material for: The Continuum of Severity of Functional Impairment Due to Indoor Air Symptoms: Prevalence and Determinants
Source: J Occup Environ Med. 2023 May 23;65(9):717–24. doi: 10.1097/JOM.0000000000002884 (PMC10487355; doi:10.1097/JOM.0000000000002884)
Supplement: SUPPLEMENTARY MATERIAL [file joem-65-0717-s002.pdf]

Supplement to 'The continuum of severity of functional impairment due to indoor air symptoms: prevalence and determinants'

Einar Eidstø, BM[1], Sanna Selinheimo, PhD[1, 2], Jussi Lampi, MD, PhD[3], Anniina Salmela, PhD[3], Juha Pekkanen, MD, PhD, prof[1, 3]

[1] Department of Public Health, Faculty of Medicine, University of Helsinki, Helsinki, Finland

[2] Finnish Institute of Occupational Health, Helsinki, Finland

[3] Department of Health Security, Environmental Health Unit, Finnish Institute for Health and Welfare, Kuopio, Finland

Methods

Participants were also asked if they had taken any measures of a predetermined list of 14 measures (Supplemental Table 4) to avoid indoor air symptoms during the past 12 months. The 14th option was "Other". Responses to the 14 measures were added up to form a sum score by giving following points to the three response options: (0 = "No", 1 = "Yes, once or twice", 2 = "Yes, repeatedly"). Those with a sum score of 0 were classified as "No avoidance", those with a sum score of 1-3 were classified as "Slight avoidance", those with a sum score of 4-6 were classified as "Moderate avoidance", and the rest with a sum score of 7 or above were classified as "Much avoidance" (Supplemental Table 5).

Supplemental Table 1. Associations of demographic characteristics with severity of indoor air-related symptoms during the past 12 months

	No symptoms (n=1347*)	Mild symptoms (n=220)	Moderate symptoms (n=142)	Severe symptoms (n=32)	Omnibus test
Sex					
Female (%)	53.7	64.1	71.7	69.7	
OR(95%CI)	1 (reference)	1.54 (1.15-2.07)	2.19 (1.50-3.19)	1.98 (0.94-4.20)	0.001
Age					
25-44y** (%)	38.5	49.8	51.7	42.4	
OR(95%CI)	1 (reference)	1.58 (1.19-2.10)	1.71 (1.21-2.41)	1.18 (0.59-2.37)	0.001
Marital status					
Married (%)	52	49.5	52.1	39.4	
OR(95%CI)	1 (reference)	0.99 (0.74-1.33)	1.11 (0.78-1.57)	0.63 (0.31-1.29)	0.56
Education					
Academic degree (%)	62.3	58.4	54.2	46.9	
OR(95%CI)	1 (reference)	1.01 (0.75-1.36)	0.87 (0.60-1.25)	0.61 (0.29-1.26)	0.51
Employment					
Employed (%)	69.7	87.4	82.6	81.8	
OR(95%CI)	1 (reference)	2.85 (1.88-4.32)	1.97 (1.26-3.10)	1.90 (0.78-4.67)	0.001
Owner-occupied housing (%)	78.6	76.1	74.3	65.6	
OR(95%CI)	1 (reference)	1.00 (0.71-1.42)	0.90 (0.60-1.37)	0.55 (0.25-1.19)	0.5
Self-reported financial situation					
At least moderately adequate***(%)	85.1	84.2	77.8	84.8	
OR(95%CI)	1 (reference)	0.93 (0.63-1.38)	0.61 (0.40-0.93)	0.97 (0.37-2.55)	0.18
Place of residence					
Urban area (%)	64.5	65.6	69.2	71.9	
OR(95%CI)	1 (reference)	0.96 (0.70-1.29)	1.10 (0.76-1.61)	1.28 (0.58-2.81)	0.86
Form of housing					
Single-family home (%)	49.2	50	44.8	43.8	
OR(95%CI)	1 (reference)	1.15 (0.86-1.53)	0.94 (0.66-1.34)	0.88 (0.43-1.80)	0.75
Smoking (%)	20.5	21.7	16.1	18.2	
OR(95%CI)	1 (reference)	1.12 (0.79-1.59)	0.79 (0.49-1.26)	0.91 (0.37-2.24)	0.66

OR (95%CI): all odds ratios besides those for sex and age are adjusted for sex and age

Omnibus test: a global test of the difference between the severity groups

*Due to missing data, numbers vary among those with no symptoms between n=1347-1371, with mild symptoms n=220-223, with moderate symptoms n=142-145, and severe symptoms n=32-33

**Compared to the reference group 45-64y

***moderately adequate, almost fully adequate, or fully adequate financial situation

Supplemental Table 2. Multivariate adjusted associations between doctor diagnosed diseases and severity of indoor air-related symptoms during the past 12 months

	No symptoms (n=1183*)	Mild symptoms (n=188)	Moderate symptoms (n=117)	Severe symptoms (n=26)	Omnibus test
**Self-reported doctor-diagnosed or treated diseases during the past 12 months**					
Sleep apnoea (%)	5.3	8.5	9.8	3.8	
OR(95%CI)	1 (reference)	2.44 (1.34-4.45)	3.26 (1.64-6.52)	0.92 (0.12-7.06)	0.002
Asthma (%)	7.3	9	20.2	32.1	
OR(95%CI)	1 (reference)	1.46 (0.84-2.55)	3.86 (2.32-6.44)	6.82 (2.92-15.92)	0.001
Allergic rhinitis (%)	15.7	30.1	39.7	55.2	
OR(95%CI)	1 (reference)	2.25 (1.57-3.21)	3.30 (2.20-4.96)	6.92 (3.19-14.99)	0.001
Atopic eczema (%)	9.8	10.5	22.6	35.7	
OR(95%CI)	1 (reference)	1.05 (0.63-1.75)	2.39 (1.47-3.89)	5.17 (2.30-11.66)	0.001
Depression (%)	8.2	5.3	6.8	21.4	
OR(95%CI)	1 (reference)	0.67 (0.34-1.34)	0.89 (0.41-1.93)	3.07 (1.16-8.17)	0.11
Irritable bowel syndrome (%)	5.7	10.1	12.4	14.8	
OR(95%CI)	1 (reference)	2.51 (1.43-4.41)	2.88 (1.51-5.48)	3.07 (1.00-9.40)	0.001
Sensitivity to poor indoor air quality (%)	3.2	9.4	24.8	69	
OR(95%CI)	1 (reference)	3.42 (1.87-6.26)	10.80 (6.12-19.07)	65.91 (27.03-160.70)	0.001
Other functional disorder** (%)	7.7	10.4	23.4	29.6	
OR(95%CI)	1 (reference)	1.77 (1.02-3.06)	4.87 (2.89-8.22)	6.70 (2.65-16.94)	0.001

OR (95%CI): Odds ratio and its 95% confidence interval adjusted for age, sex, employed/studying, education, marital status

Omnibus test: a global test of the difference between the severity groups

*Due to missing data, numbers vary among those with no symptoms between n=1183-1209, with mild symptoms n=188-193, with moderate symptoms n=117-126, and severe symptoms n=26-29

**chronic fatigue syndrome, fibromyalgia, chronic pain syndrome, odour sensitivity, multiple chemical sensitivity, noise sensitivity, and electromagnetic sensitivity

Supplemental Table 3. Multivariate adjusted associations of health, ability to work, quality of life, and environmental sensitivities with severity of indoor air-related symptoms during the past 12 months

	No symptoms (n=1344*)	Mild symptoms (n=218)	Moderate symptoms (n=141)	Severe symptoms (n=32)	Omnibus test
**Self-reported health**					
Good or fairly good (%)	79.8	82.8	74.8	56.3	
OR (95%CI)	1 (reference)	0.83 (0.56-1.23)	0.56 (0.36-0.87)	0.20 (0.09-0.43)	<0.001
**Self-reported ability to work**					
Good or very good (%)	75.7	82	72.5	59.4	
OR (95%CI)	1 (reference)	0.90 (0.61-1.34)	0.49 (0.32-0.76)	0.28 (0.13-0.62)	<0.001
**Self-reported quality of life**					
Good or very good (%)	83.9	83.8	76.9	50	
OR (95%CI)	1 (reference)	0.72 (0.48-1.07)	0.47 (0.30-0.73)	0.13 (0.06-0.29)	<0.001
**Self-perceived sensitivity to experience symptoms** from**					
Odours (%)	7.7	16.4	27.7	42.4	
OR (95%CI)	1 (reference)	2.71 (1.75-4.18)	5.35 (3.43-8.35)	10.20 (4.79-21.71)	<0.001
Chemicals (%)	6.2	9.6	23.8	50	
OR (95%CI)	1 (reference)	2.08 (1.21-3.56)	6.23 (3.84-10.10)	21.55 (9.80-47.38)	<0.001
Poor indoor air quality (%)	6.9	15.5	37.1	87.9	
OR (95%CI)	1 (reference)	2.60 (1.67-4.02)	7.79 (5.11-11.88)	99.63 (33.73-294.30)	<0.001
Noise (%)	5.7	11.9	20.6	24.2	
OR (95%CI)	1 (reference)	2.39 (1.47-3.90)	4.26 (2.60-6.99)	5.18 (2.20-12.19)	<0.001

OR (95%CI): Odds ratio and its 95% confidence interval adjusted for age, sex, employed/studying, education, marital status

Omnibus test: a global test of the difference between the severity groups

*Due to missing data, numbers vary among those with no symptoms between n=1344-1362, with mild symptoms n=218-222, with moderate symptoms n=141-143, and severe symptoms n=32-33

**Much or very much more easily than other people

Supplemental Table 4. The prevalence of various measures to avoid symptoms in the past 12 months

	**No symptoms (n=1362*), %**	**Symptoms, no functional impairment (n=83), %**	**Symptoms, mild functional impairment (n=190), %**	**Symptoms, moderate functional impairment (n=89), %**	**Symptoms, severe functional impairment (n=29), %**
**Measures** to avoid symptoms (past 12 months)**					
More cleaning	0	28.9	43.5	46.1	74.2
Renovating the home	0	10.7	12.4	16.9	23.3
Adjusting the ventilation at home	0	26.2	30.6	33.7	51.7
Disposal, disinfection, or ozonization of movables	0	9.6	7.3	11.1	23.3
Sleeping on the balcony	0	0	0.5	2.2	3.3
Moving to another apartment	0	2.4	2.1	6.6	16.7
Changing the interior of the workplace	0	7.2	16.1	23.3	23.3
Avoiding certain spaces at work/school	0	25.3	42.1	64.1	66.7
Working/studying from home	0	8.3	10.3	15.4	20
Changing jobs	0	2.4	7.2	10	26.7
Avoiding public spaces	0	3.6	4.7	16.3	20
Giving up a hobby	0	0	0.5	18.5	23.3
The use of vitamins/supplements or a change of diet	0	9.6	23.4	41.8	61.3

*Due to missing data, numbers vary among those with no symptoms n=1362, no functional impairment n=83-84, mild functional impairment n=190-195, moderate functional impairment n=89-92, severe functional impairment n=29-31
**At least once or twice

Supplemental Table 5. Multivariate adjusted associations between determinants and the amount of avoidance

	No avoidance (n=1411*)	Slight avoidance (n=191)	Moderate avoidance (n=81)	Much avoidance (n=50)	Omnibus test
**Self-reported health**					
Good or fairly good (%)	80.2	81.2	69.1	64.7	
OR(95%CI)	1 (reference)	0.73 (0.48-1.10)	0.33 (0.19-0.56)	0.32 (0.17-0.60)	<0.001
**Self-reported ability to work**					
Good or very good (%)	76.3	80.8	63	68.6	
OR(95%CI)	1 (reference)	0.81 (0.53-1.22)	0.29 (0.17-0.48)	0.43 (0.22-0.84)	<0.001
**Self-reported quality of life**					
Good or very good (%)	83.9	84	69.1	64.7	
OR(95%CI)	1 (reference)	0.7 (0.46-1.08)	0.27 (0.16-0.46)	0.27 (0.14-0.52)	<0.001
**Sensitivity to experience symptoms** from**					
Odors (%)	8.1	19.8	26.8	36	
OR(95%CI)	1 (reference)	3.14 (2.05-4.82)	4.50 (2.59-7.82)	6.82 (3.62-12.85)	<0.001
Chemicals (%)	6.2	13.4	21	44	
OR(95%CI)	1 (reference)	3.14 (1.91-5.16)	4.82 (2.59-8.97)	14.70 (7.72-28.01)	<0.001
Poor indoor air quality (%)	6.8	21.8	46.3	62	
OR(95%CI)	1 (reference)	3.66 (2.40-5.57)	12.01 (7.26-19.88)	20.88 (11.17-39.03)	<0.001
Noise (%)	5.7	14.1	22	30	
OR(95%CI)	1 (reference)	2.84 (1.74-4.65)	5.14 (2.85-9.29)	7.39 (3.78-14.45)	<0.001
**Self-reported doctor-diagnosed or treated diseases (past 12 months)**	n=1258***	n=164	n=73	n=37	
Asthma (%)	7.7	12.7	17.8	19.5	
OR(95%CI)	1 (reference)	1.84 (1.09-3.12)	2.86 (1.50-5.46)	3.17 (1.41-7.15)	<0.001
Allergic rhinitis (%)	16	32.4	39	62.5	
OR(95%CI)	1 (reference)	2.34 (1.62-3.38)	3.30 (2.00-5.45)	7.95 (4.06-15.57)	<0.001
Atopic eczema (%)	9.8	15.9	15.1	33.3	
OR(95%CI)	1 (reference)	1.63 (1.02-2.60)	1.57 (0.80-3.09)	4.36 (2.16-8.82)	<0.001
Depression (%)	7.9	6.1	9.3	16.2	
OR(95%CI)	1 (reference)	0.84 (0.42-1.68)	1.43 (0.62-3.28)	2.36 (0.92-6.04)	0.29

OR (95%CI): Odds ratio and its 95% confidence interval adjusted for age, sex, employed/studying, education, marital status

Omnibus test: a global test of the difference between the avoidance groups

*Due to missing data, numbers vary among those with no avoidance between n=1411-1432, with sligth avoidance between n=191-194, with moderate avoidance between n=81-82, and with much avoidance between n=50-51

**Much or very much more easily than other people

***Due to missing data, numbers vary among those with no avoidance between n=1258-1265, with sligth avoidance between n=164-173, with moderate avoidance between n=73-77, and with much avoidance between n=37-41
