# Supplementary material for: The Continuum of Severity of Functional Impairment Due to Indoor Air Symptoms: Prevalence and Determinants
Source: J Occup Environ Med. 2023 May 23;65(9):717–24. doi: 10.1097/JOM.0000000000002884 (PMC10487355; doi:10.1097/JOM.0000000000002884)
Supplement: SUPPLEMENTARY MATERIAL [file joem-65-0717-s001.docx]

Abstract

**Objective**: To study functional impairment due to indoor air-related symptoms and associated characteristics.
**Methods:** A questionnaire survey of a random sample of Finnish people aged 25-64. Analyses were done using multivariate multinomial logistic regression.
**Results:** 23.1% reported indoor air-related symptoms, 1.8% severe functional impairment due to symptoms, 5.3% moderate, 11.1% mild, and 4.9% reported no impairment. Those with severe functional impairment showed the strongest associations with comorbid diseases, e.g. asthma and irritable bowel syndrome, perceived sensitivities to several environmental factors, like chemicals, and likelihood of having symptoms in multiple organs, while those with no or little functional impairment showed weak or even inverse associations. Similar results emerged with severity of indoor air-related symptoms.
**Conclusions:** Individuals with indoor air-related symptoms are a very heterogeneous group. This should be better considered in future research and clinical practice.

**Keywords**: indoor air, symptoms, functional impairment, disability, population-based survey, epidemiology, comorbid diseases, socioeconomic factors, idiopathic environmental intolerance

Learning outcomes

- To be able to describe the prevalence of different levels of functional impairment due to indoor air-related symptoms among the adult population and associated factors.
- To understand the importance of assessing the differences in functional impairment due to indoor air-related symptoms.
- To remember to consider in their own clinical practice that people with more severe functional impairment due to indoor air-related symptoms also have more somatic, psychiatric, and functional comorbidities, which may prevent them from gaining from care and support.

**Introduction**

Indoor air or building-related symptoms are common in non-industrial environments [1-5]. Symptoms range from organ-specific, such as mucous-membrane irritation and dry skin to general, non-specific symptoms of fatigue and headaches [6]. Risk factors for indoor air-related symptoms include not only indoor air pollution, such as moulds, chemicals, and poor ventilation, but also individual factors, such as sex, concurrent diseases, personality traits, and psychosocial factors at work [3, 7-10]. In addition to increasing symptoms, mould and dampness exposure has been shown to be associated with onset of new asthma also in adults [11, 12].

A few clinical studies have shown that some of the patients have difficult functional impairment due to persistent symptoms, meaning severe restrictions to personal, occupational and social life. These persistent symptoms have been shown to be associated with limitations of daily activities due to e.g. avoidance of buildings that the individual perceives to trigger symptoms. Also, adverse effects on work ability appear to be common among these individuals. In the most severe cases, disability to work persists despite environmental repairs, improvements in indoor air quality or avoidance of the building in question [13-18]. These persistent indoor air-related symptoms have been shown to affect quality of life even more than common diseases such as asthma or depression [19]. However, the great majority of studies on indoor air-related symptoms do not consider the severity of symptoms or associated levels of functional impairment, but typically only symptom frequency [2, 20-22].

While symptoms and symptom severity are important for diagnosis, assessing functional capacity is also important in clinical practice. In addition to a biomedical model that focuses on the underlying disease, functional capacity considers the individual´s psychological, social and physical abilities to perform in daily life [23]. Hence, this study focuses on levels of functional impairment. Assessing the level of functional impairment helps to identify, design and target the support according to the individual´s needs, such as job accommodations, sick leave, or other social support and rehabilitation. In research, studying the levels of functional impairment helps to determine more accurately the burden of different symptoms and diseases to society [24] and thereby helps to guide resource allocation in health care and public health policy. In addition, estimates of the prevalence of symptoms are not comparable between studies without considering the severity of symptoms or of functional impairment. One population-based study has been done among pregnant women on level of functional impairment due to environmental intolerance [25]. However, to our knowledge, there are no previous population-based studies that would have explored the full range of the variability in the level of functional impairment, or of symptom severity, due to indoor air-related symptoms.

Therefore, the present study determined for the first time the prevalence of different levels of functional impairment due to indoor air-related symptoms in a representative, population-based sample. Furthermore, this study explored the associations of sociodemographic, clinical, and other characteristics with levels of functional impairment. Analyses were repeated also for severity of indoor air-related symptoms.

**Methods**

*Study population*
The National Survey on Indoor Air was conducted jointly by the Finnish Institute for Health and Welfare, and the University of Helsinki from November 2018 to March 2019. A postal questionnaire was sent to a random sample of 4997 25-64-year-old Finnish speakers in Finland, excluding Åland. 1797 (36%) subjects responded either to the postal or the electronic questionnaire.

The study was approved by an Institutional Review Board (IRB) of the Finnish Institute for Health and Welfare.

*Health outcomes and variable coding*
Respondents were asked, “Have you ever gotten symptoms from indoor air at home?”. The question had three options (No symptoms; yes, during the past 12 months; yes, over 12 months ago). The same question was then asked about indoor air symptoms at work. Those who reported symptoms during the past 12 months either at home or at work were considered symptomatic and those who reported no symptoms or had had symptoms only over 12 months ago were considered non-symptomatic. Those with missing answers to these questions were classified as missing and excluded from further analyses.

Those individuals who reported symptoms were further asked “Have these symptoms made it difficult to work, manage your domestic responsibilities, or get along with other people during the past 12 months?” There were five response options (not at all, quite a little, moderately, quite a lot, a lot). For the analysis, the last two categories were combined, as there were only 8 respondents in the last category, to form the following 5 groups: no symptoms, symptoms with no functional impairment, mild functional impairment, moderate functional impairment, and severe functional impairment.

In addition to functional impairment, respondents were asked about the self-perceived severity of indoor air-related symptoms and avoidance behaviour (see Methods, Supplemental Digital Content 1, <http://links.lww.com/JOM/B345>). Self-perceived severity of symptoms was asked on a 4-point scale (mild, moderate, severe, very severe symptoms). Due to low numbers, the last two categories were combined to form 4 groups: no symptoms, mild symptoms, moderate symptoms, and severe symptoms. Participants were also asked about the measures to avoid indoor air symptoms they had taken during the past 12 months (see Table 4, Supplemental Digital Content 1, <http://links.lww.com/JOM/B345>).

The respondents were also asked “Which symptoms have you had due to indoor air and how often in the last 12 months?” They were given a predetermined list of 18 symptoms, and they had five response options from “Never” to “Nearly every day”. In the analysis, symptoms were dichotomised (At least once or twice a week *vs* Less often). The 18 different symptoms asked were classified into 5 groups. The group of symptoms was coded to be present, if at least one of the symptoms in the group was present. The group ‘respiratory and eye symptoms’ included 5 symptoms (nasal congestion, hoarse voice, shortness of breath, cough, eye symptoms,), ‘skin symptoms’ were asked with only one question, ’joint symptoms’ included joint symptoms and pricking of limbs, ‘general symptoms’ included 5 symptoms (headache, vertigo, nausea, fatigue, brain fog), and ‘other symptoms’ included 5 symptoms (heart palpitations, perspiration, urinary incontinence, diarrhoea, fever).

Participants were asked to estimate their health on a 5-point scale from “Good” to “Bad”. In the analysis, health was dichotomised (Good or fairly good *vs* Average, fairly bad or Bad). Similarly, the quality-of-life question had five options from “Very bad” to “Very good”, and it was dichotomised (Very good or good *vs* Average, bad or very bad). Ability to work was estimated by The Work Ability score (WAS) [26] This one-item scale consists of a worker’s self-assessment of his/her current ability compared to the lifetime best. It ranges from 0 (“No ability to work”) to 10 (“Ability to work at its best”). In this study, we dichotomised it into poor – moderate work ability (0-7 points) and good to excellent work ability (8-10 points). The prevalence of eleven common diseases such as asthma or coronary artery disease and nine functional disorders was assessed with the question “During the past 12 months, have you had any of these health conditions diagnosed or treated by a physician?”. The respondent was coded as having an “Other functional disorder”, if they reported having one or more of the following: chronic fatigue syndrome, fibromyalgia, chronic pain syndrome, odour sensitivity, multiple chemical sensitivity, noise sensitivity, or electromagnetic sensitivity [27-29]. Self-perceived sensitivity to odours, chemicals, poor indoor air quality, and noise was assessed with the question “Do you think that you get symptoms more easily than other people when exposed to the following factors?”. The question had 4 response options (No; Yes, a little more easily; Yes, much more easily; Yes, very much more easily). The question was dichotomized (Much or very much more easily *vs* No or little more easily). Participants were asked “Do you smoke?” They had seven options from “Not at all” to “Daily, over 15 cigarettes a day”. In the analysis, smoking was recategorized into those that never smoked and those that at least sometimes smoked.

*Demographic characteristics and variable coding*

Respondents were asked whether they were living in “owner-occupied housing”, “a rented apartment”, “a housing cooperative or similar”, or “somewhere else”. In the analysis, this was dichotomised (Owner-occupied housing *vs* Other). Financial situation was assessed with the question “Do you have enough money in view of your needs?” There were five options (Not at all, A little, Moderately adequate, Almost fully adequate, Fully adequate). In the analysis, financial situation was dichotomised (Poor or very poor *vs* At least moderately adequate). The respondents were given four options to describe their place of residence (Inner city, Suburbs, Countryside population centre, Dispersed settlement). Place of residence was dichotomised (Urban area *vs* Countryside). Form of housing had four options (Single-family home, Semi-detached house or terraced house, Apartment building, Somewhere else). In the analysis, form of housing was dichotomised (Single-family home *vs* Other).

Due to data confidentially issues, age was available only in 5-year categories, and it was treated as a continuous variable in the analyses. Employment status was dichotomised into “Employed or studying” and “Others”, education into “Academic degree” and “No academic degree”, and marital status into “Married” and “Not married”.

**Statistical analysis**

Multivariate models were run with multinomial logistic regression. The main dependent variable used was functional impairment due to indoor air-related symptoms, but most analyses were repeated also for severity of indoor-air related symptoms and avoidance behaviour. Analyses were adjusted for age, sex, employed/studying, education, and marital status – though in the analyses of Table 2, only sex and age were used as confounders. The results are presented as odds ratios (ORs), including 95% confidence intervals (CI). In addition, an omnibus test, a global test of the differences between all of the functional impairment groups, is reported. Sensitivity analyses were conducted by analyzing separately those who reported symptoms only at home or at work.

Statistical analyses were conducted in IBM SPSS 28.0 for Windows (SPSS Illinois, Chicago, Illinois, USA).

**Results**
Of the respondents, 23.1% experienced indoor air-related symptoms, but only 18.2% reported functional impairment due to symptoms (Figure 1). Most experienced mild functional impairment (11.1%), 5.3% moderate, and 1.8% severe functional impairment from indoor air-related symptoms. Of the respondents, 4.9% reported no functional impairment despite symptoms.

Severity of indoor air-related symptoms and amount of avoidance behaviour due to these symptoms were strongly associated with severity of functional impairment (Table 1). About two-thirds of the subjects had the same degree of severity in both functional impairment and in symptom severity. Strong associations were also seen with visiting a physician and being on sick leave: while only 2.3% of those with symptoms, but no functional impairment, had visited a physician due to symptoms and none had been on sick leave, 53.1% of those with severe functional impairment had visited a physician and been on sick leave. While only a small portion of those with no functional impairment reported measures to avoid symptoms, a much larger portion of those with severe functional impairment did. Changing jobs and giving up a hobby had the largest relative increase with increasing functional impairment (Table 4, Supplemental Digital Content 1, <http://links.lww.com/JOM/B345>).

Those with no functional impairment, mild functional impairment, or moderate functional impairment were significantly younger than the respondents without symptoms (Table 2). Especially those with moderate functional impairment were more often female and less often married. Particularly those with mild functional impairment were more often employed than the other groups. Respondents with moderate or severe functional impairment lived less often in owner-occupied housing and reported more often poorer financial situation. Smoking, place of residence, and form of housing had no significant associations with different levels of functional impairment.

Self-reported doctor-diagnosed allergic diseases, sensitivity to poor indoor air quality, irritable bowel syndrome, and other functional disorders were more common especially among those with severe functional impairment, but also among those with mild to moderate functional impairment (Table 3). Depression tended to be less common among those with no functional impairment, whereas it was significantly more common only among those with severe functional impairment.

Those with moderate and especially those with severe functional impairment reported poorer health, poorer ability to work, and lower quality of life (Table 4) than other respondents. Interestingly, those with no functional impairment despite symptoms had better self-reported health than respondents without symptoms. The prevalence of self-perceived sensitivity to environmental factors, such as odours, chemicals, poor indoor air quality, and noise, increased strongly with exacerbating severity of functional impairment when compared to those without symptoms.

Using severity of symptoms (Tables 1-3, Supplemental Digital Content 1, <http://links.lww.com/JOM/B345>) instead of functional impairment (Tables 2-4) due to indoor air-related symptoms yielded very similar associations, although severity of functional impairment showed in general slightly stronger associations than severity of symptoms. This was true especially for self-reported financial situation (Table 2), sleep apnoea, depression, irritable bowel syndrome and other functional disorders (Table 3), and self-reported health, ability to work and quality of life (Table 4), whereas asthma, atopic eczema (Table 3), and sensitivity to experience symptoms from odours, chemicals, or poor indoor air quality (Table 4) were somewhat more strongly associated with severity of symptoms. Also, avoidance behaviour produced mostly similar, but weaker associations (see Table 5, Supplemental Digital Content 1, <http://links.lww.com/JOM/B345>) than functional impartment or severity of symptoms. However, amount of avoidance behaviour had no significant association with depression.

Those with no or mild functional impairment reported mainly respiratory and eye symptoms, whereas among those with severe functional impairment, general symptoms were almost as common as respiratory and eye symptoms (Figure 2). The prevalence of all groups of symptoms increased with increasing functional impairment. The symptoms with the strongest relative increases were joint symptoms, general symptoms, and other symptoms.

As sensitivity analyses, we also explored associations separately for those with indoor air-related symptoms at work and at home (data not shown). The small number of those with symptoms at home (n=116), especially in the moderate (n=30) and severe functional impairment (n=14) groups, made the associations unstable. The main differences between those with symptoms at home compared to those with symptoms at work were that those with symptoms at home were less often employed, married, or had an adequate financial situation and had more often depression. This was true particularly among those with severe functional impairment. The associations for those with symptoms at work were very similar to those presented in Tables 2-4.

**Discussion**

The present population-based study shows that subjects reporting indoor air-related symptoms are a heterogeneous group. Two-thirds of the individuals with indoor air-related symptoms report at most mild functional impairment, i.e., difficulties with work, managing domestic responsibilities, or social life, due to indoor air-related symptoms. The prevalence of comorbid diseases, self-perceived sensitivities to environmental factors, symptoms in multiple organs, and to some extent poorer financial situation were more common among those with more severe functional impairment, whereas the associations were weaker or even inverse among those with no or mild functional impairment. Similar results were seen with severity of indoor air-related symptoms.

The present study is, to our knowledge, the first study exploring the levels of functional impairment and symptom severity due to indoor-air related symptoms in a representative population-based sample. Functional impairment among those with indoor air-related symptoms has earlier been explored in a few small-scale or qualitative [13, 30-33] and quantitative [14-18] studies and have therefore focused on those with more severe symptoms. Two prospective studies [16, 17] have shown that a higher number of symptoms is associated with poorer work ability at follow-up.

Individuals with indoor air-related symptoms have been shown to have comorbidity with several diseases, both somatic and psychiatric [3, 20, 34]. Our study is the first to show that the prevalence of comorbidities increases along with the level of functional impairment due to indoor air-related symptoms in the population. For example, while 10.8% of those with mild functional impairment had asthma, compared to 7.3% of individuals without symptoms, a quarter of those with severe functional impairment had asthma. The latter estimate is consistent with the estimate from a previous population-based study from Finland [20]. Earlier studies based on data from occupationally active individuals have shown that those with indoor air-related symptoms have depression more often than non-symptomatic individuals [3, 35]. We, however, found that depression was more common only among those with severe functional impairment, whereas the prevalence was decreased among those with no or mild functional impairment.

We observed that increasing severity of functional impairment due to indoor air-related symptoms was associated with an increased risk of reporting intolerance not only to indoor air but also to other environmental exposures. This finding is consistent with earlier findings from population-based studies in Finland [25] and Sweden [36] showing strong overlap between different idiopathic environmental intolerances, conditions that are characterized by symptoms from multiple organ systems attributed to various environmental factors that are tolerated by the majority of people [37]. As observed before for environmental intolerance [25], also in the present study the likelihood of having symptoms from multiple organs increased with increasing severity of functional impairment. We further observed a similar increased risk of having disorders that are labelled as functional somatic syndromes, like irritable bowel syndrome and chronic fatigue syndrome, with increasing severity of functional impairment [27]. These findings are consistent with earlier suggestions, that there is substantial overlap between the functional somatic syndromes or manifestations of persistent physical symptoms [27] which may result from shared predisposing and perpetuating factors and common underlying mechanisms. E.g. controlled studies done among those with increased sensitivity to electromagnetic fields [38] and multiple chemicals [39] have shown that the symptoms among these individuals are not due to the suspected exposures, but knowledge or suspicion of being exposed. There is also extensive evidence that, in addition to indoor air impurities and dampness and mould [12, 40-42], indoor air-related symptoms are associated with other biopsychosocial factors as well, such as gender, stress, lack of social support, anxiety, and job demands [2, 3, 7, 9, 40, 43], that may serve as perpetuating factors for functional impairment. In addition, we observed in the present study that the number of comorbidities was increased especially among those with severe functional impairment. Taken together, these observations suggest that factors other than indoor air impurities may play a larger aetiological role among those with more severe functional impairment, which is also consistent with the clinical experience.

Those with lower socioeconomic status have been shown to have more diseases, lower self-perceived health, and higher mortality [44]. However, no previous study has shown similar associations with indoor air-related symptoms. Our findings indicate that the more functional impairment due to indoor air symptoms one has, the more likely one is to report a worse financial situation. This may be due to inability to work because of symptoms. Indeed, self-reported ability to work was significantly lower among those with moderate and severe functional impairment (Table 3). Although the causality of the factors cannot be assessed, our results suggest that those with more functional impairment may be financially in a disadvantaged position compared to those with mild or no symptoms. As a similar trend was not seen for education, which is another commonly used indicator of socioeconomic status, more research is clearly needed on the strength and origins of the socioeconomic gradient in indoor air-related health and on the best ways to reduce this gradient.

In the present study, severity of symptoms was strongly related to severity of functional impairment due to these symptoms. Also, almost all the observed results were very similar when using either severity of symptoms or functional impairment as the outcome. However, the two groups, while overlapping by about two-thirds, were not the same and, in general, stronger associations were observed with functional impairment than with severity of symptoms. Compared to those with severe functional impairment, those with severe symptoms reported better health, better ability to work, or better quality of life. Possibly due to these reasons, fewer of those with severe symptoms reported being in a poor financial situation. Also, severe functional impairment was clearly more strongly associated with depression. These differences support the importance of assessing the functional impairment of the patient in a clinical setting and in research, in addition to severity of symptoms.

One of the major strengths of this study is that it is the first population-based study describing different levels of functional impairment due to indoor air-related symptoms. The participation rate was only 36%. However, as no participation bias was detected for indoor air-related symptoms [45] when compared to a previous study with a higher participation rate [46], the sample can be considered suitable for the present purpose. This study also had several limitations. The data was self-reported, and no clinical measurements were performed to assess the level of functional impairment or the presence of chronic diseases. We also did not have information on the respondents’ income. Another limitation of the study is its cross-sectional study design, which precludes the assessment of causality. In general, the direction of causality of the observed associations is difficult to assess. Indoor air-related symptoms may worsen the psychosocial burden, e.g. increase stress, but psychosocial factors may also increase symptom reports. More longitudinal studies are therefore urgently needed [47, 48].

In conclusion, the present results show that the degree of functional impairment due to indoor air-related symptoms is a continuum in the population, and that individuals with indoor air-related symptoms are a very heterogeneous group. When trying to understand this heterogeneity, other biological, psychological, and social factors [2, 3, 7, 40] need to be considered in addition to indoor air quality. The majority of those with symptoms have at most mild functional impairment and differ less from those with no symptoms. The more severe functional impairment one has, the more likely one is to have comorbidities, self-reported environmental sensitivities, symptoms in multiple organs, and possibly a poorer financial situation. Similar results were seen with severity of symptoms. Due to this heterogeneity, the determinants and aetiologies of symptoms and functional impairments of differing severity likely differ and thus there is also a need for different types of treatments and support. Therefore, the heterogeneity of indoor air-related symptoms should be better considered in future studies on indoor air and health, and clinical practice.

**References**

1. Bluyssen, P.M., Roda, C., Mandin, C., et al., *Self-reported health and comfort in 'modern' office buildings: first results from the European OFFICAIR study.* Indoor Air, 2016. **26**(2): p. 298-317.

2. Runeson-Broberg, R. and Norback, D., *Sick building syndrome (SBS) and sick house syndrome (SHS) in relation to psychosocial stress at work in the Swedish workforce.* Int Arch Occup Environ Health, 2013. **86**(8): p. 915-22.

3. Magnavita, N., *Work-related symptoms in indoor environments: a puzzling problem for the occupational physician.* Int Arch Occup Environ Health, 2015. **88**(2): p. 185-96.

4. Selinheimo, S., Lampi, J., and Pekkanen, J., *Parent's self-reported indoor environment-related symptoms and health worry increase symptom reports among their children at school-Study in two independent populations.* Indoor Air, 2021. **31**(5): p. 1298-1307.

5. Park, J. and Gilmour, H., *Medically unexplained physical symptoms (MUPS) among adults in Canada: Comorbidity, health care use and employment.* Health Rep, 2017. **28**(3): p. 3-8.

6. Redlich, C.A., Sparer, J., and Cullen, M.R., *Sick-building syndrome.* Lancet, 1997. **349**(9057): p. 1013-6.

7. Azuma, K., Ikeda, K., Kagi, N., Yanagi, U., and Osawa, H., *Evaluating prevalence and risk factors of building-related symptoms among office workers: Seasonal characteristics of symptoms and psychosocial and physical environmental factors.* Environ Health Prev Med, 2017. **22**(1): p. 38.

8. Norback, D., *An update on sick building syndrome.* Curr Opin Allergy Clin Immunol, 2009. **9**(1): p. 55-9.

9. Bakke, J.V., Moen, B.E., Wieslander, G., and Norback, D., *Gender and the physical and psychosocial work environments are related to indoor air symptoms.* J Occup Environ Med, 2007. **49**(6): p. 641-50.

10. Brasche, S., Bullinger, M., Morfeld, M., Gebhardt, H.J., and Bischof, W., *Why do women suffer from sick building syndrome more often than men?--subjective higher sensitivity versus objective causes.* Indoor Air, 2001. **11**(4): p. 217-22.

11. Norback, D., Zock, J.P., Plana, E., et al., *Mould and dampness in dwelling places, and onset of asthma: the population-based cohort ECRHS.* Occup Environ Med, 2013. **70**(5): p. 325-31.

12. Hurrass, J., Heinzow, B., Aurbach, U., et al., *Medical diagnostics for indoor mold exposure.* Int J Hyg Environ Health, 2017. **220**(2 Pt B): p. 305-328.

13. Vuokko, A., Karvala, K., Suojalehto, H., et al., *Clinical Characteristics of Disability in Patients with Indoor Air-Related Environmental Intolerance.* Saf Health Work, 2019. **10**(3): p. 362-369.

14. Al-Ahmad, M., Manno, M., Ng, V., Ribeiro, M., Liss, G.M., and Tarlo, S.M., *Symptoms after mould exposure including Stachybotrys chartarum, and comparison with darkroom disease.* Allergy, 2010. **65**(2): p. 245-55.

15. Edvardsson, B., Bergdahl, J., Eriksson, N., and Stenberg, B., *Coping and self-image in patients with symptoms attributed to indoor environment.* Arch Environ Occup Health, 2013. **68**(3): p. 145-52.

16. Edvardsson, B., Stenberg, B., Bergdahl, J., Eriksson, N., Linden, G., and Widman, L., *Medical and social prognoses of non-specific building-related symptoms (Sick Building Syndrome): a follow-up study of patients previously referred to hospital.* Int Arch Occup Environ Health, 2008. **81**(7): p. 805-12.

17. Karvala, K., Nordman, H., Luukkonen, R., and Uitti, J., *Asthma related to workplace dampness and impaired work ability.* Int Arch Occup Environ Health, 2014. **87**(1): p. 1-11.

18. Karvala, K., Uitti, J., Luukkonen, R., and Nordman, H., *Quality of life of patients with asthma related to damp and moldy work environments.* Scand J Work Environ Health, 2013. **39**(1): p. 96-105.

19. Selinheimo, S., Vuokko, A., Hublin, C., et al., *Health-related quality among life of employees with persistent nonspecific indoor-air-associated health complaints.* J Psychosom Res, 2019. **122**: p. 112-120.

20. Karvala, K., Sainio, M., Palmquist, E., Claeson, A.S., Nyback, M.H., and Nordin, S., *Building-Related Environmental Intolerance and Associated Health in the General Population.* Int J Environ Res Public Health, 2018. **15**(9).

21. Zhang, X., Sahlberg, B., Wieslander, G., Janson, C., Gislason, T., and Norback, D., *Dampness and moulds in workplace buildings: associations with incidence and remission of sick building syndrome (SBS) and biomarkers of inflammation in a 10 year follow-up study.* Sci Total Environ, 2012. **430**: p. 75-81.

22. Eriksson, N.M. and Stenberg, B.G., *Baseline prevalence of symptoms related to indoor environment.* Scand J Public Health, 2006. **34**(4): p. 387-96.

23. Cieza, A., Sabariego, C., Bickenbach, J., and Chatterji, S., *Rethinking Disability.* BMC Med, 2018. **16**(1): p. 14.

24. Vos, T., *"The case against annual profiles for the valuation of disability weights"*, in *Summary measures of population health: concepts, ethics, measurement and applications*, Murray, C.J., et al., Editors. 2002, World Health Organization.

25. Vuokko, A., Karvala, K., Lampi, J., et al., *Environmental Intolerance, Symptoms and Disability Among Fertile-Aged Women.* Int J Environ Res Public Health, 2018. **15**(2).

26. Gould, R., Ilmarinen, J., Järvisalo, J., and Koskinen, S., *Dimensions of work ability. Results from the Health 2000 Survey.* 2008, Finnish Institute of Occupational Health: Helsinki (Finland). p. 25-34.

27. Henningsen, P., Zipfel, S., Sattel, H., and Creed, F., *Management of Functional Somatic Syndromes and Bodily Distress.* Psychother Psychosom, 2018. **87**(1): p. 12-31.

28. Barsky, A.J. and Borus, J.F., *Functional somatic syndromes.* Ann Intern Med, 1999. **130**(11): p. 910-21.

29. Dantoft, T.M., Nordin, S., Andersson, L., Petersen, M.W., Skovbjerg, S., and Jorgensen, T., *Multiple chemical sensitivity described in the Danish general population: Cohort characteristics and the importance of screening for functional somatic syndrome comorbidity-The DanFunD study.* PLoS One, 2021. **16**(2): p. e0246461.

30. Soderholm, A., Ohman, A., Stenberg, B., and Nordin, S., *Experience of living with nonspecific building-related symptoms.* Scand J Psychol, 2016. **57**(5): p. 406-12.

31. Finell, E., Seppala, T., and Suoninen, E., *"It Was Not Me That Was Sick, It Was the Building": Rhetorical Identity Management Strategies in the Context of Observed or Suspected Indoor Air Problems in Workplaces.* Qual Health Res, 2018. **28**(8): p. 1366-1377.

32. Finell, E. and Seppala, T., *Indoor air problems and experiences of injustice in the workplace: A quantitative and a qualitative study.* Indoor Air, 2018. **28**(1): p. 125-134.

33. Vuokko, A., Selinheimo, S., Sainio, M., et al., *Decreased work ability associated to indoor air problems--An intervention (RCT) to promote health behavior.* Neurotoxicology, 2015. **49**: p. 59-67.

34. Claeson, A.S., Andersson, H., Wikdahl, F., Nyback, M.H., and Nordin, S., *Comorbidity of Airway Inflammatory Diseases in Chemical and Building-Related Intolerance.* J Occup Environ Med, 2018. **60**(4): p. 295-300.

35. Kinman, G. and Griffin, M., *Psychosocial factors and gender as predictors of symptoms associated with sick building syndrome.* Stress and Health, 2008. **24**(2): p. 165-171.

36. Palmquist, E., Claeson, A.S., Neely, G., Stenberg, B., and Nordin, S., *Overlap in prevalence between various types of environmental intolerance.* Int J Hyg Environ Health, 2014. **217**(4-5): p. 427-34.

37. IPCS/WHO, *Conclusions and recommendations of a workshop on multiple chemical sensitivities (MCS).* Regulatory toxicology and pharmacology, 1996. **24**: p. 188-189.

38. Rubin, G.J., Hillert, L., Nieto-Hernandez, R., van Rongen, E., and Oftedal, G., *Do people with idiopathic environmental intolerance attributed to electromagnetic fields display physiological effects when exposed to electromagnetic fields? A systematic review of provocation studies.* Bioelectromagnetics, 2011. **32**(8): p. 593-609.

39. Das-Munshi, J., Rubin, G.J., and Wessely, S., *Multiple chemical sensitivities: A systematic review of provocation studies.* J Allergy Clin Immunol, 2006. **118**(6): p. 1257-64.

40. Sakellaris, I., Saraga, D., Mandin, C., et al., *Association of subjective health symptoms with indoor air quality in European office buildings: The OFFICAIR project.* Indoor Air, 2021. **31**(2): p. 426-439.

41. Azuma, K., Ikeda, K., Kagi, N., Yanagi, U., and Osawa, H., *Physicochemical risk factors for building-related symptoms in air-conditioned office buildings: Ambient particles and combined exposure to indoor air pollutants.* Sci Total Environ, 2018. **616-617**: p. 1649-1655.

42. Sun, Y., Hou, J., Cheng, R., Sheng, Y., Zhang, X., and Sundell, J., *Indoor air quality, ventilation and their associations with sick building syndrome in Chinese homes.* Energy and Buildings, 2019. **197**: p. 112-119.

43. Marmot, A.F., Eley, J., Stafford, M., Stansfeld, S.A., Warwick, E., and Marmot, M.G., *Building health: an epidemiological study of "sick building syndrome" in the Whitehall II study.* Occup Environ Med, 2006. **63**(4): p. 283-9.

44. Mackenbach, J.P., Stirbu, I., Roskam, A.J., et al., *Socioeconomic inequalities in health in 22 European countries.* N Engl J Med, 2008. **358**(23): p. 2468-81.

45. Pekkanen, J., Jousilahti, P., and Tiina, L., *Indoor air-associated symptoms (in Finnish)*, in *Health, functional capacity and welfare in Finland – FinHealth 2017 study*, Koponen, P., et al., Editors. 2018, National Institute for Health and Welfare: Helsinki. p. 90-91.

46. Borodulin, K. and Sääksjärvi, K., Editors, *FinHealth 2017 Study – Methods*. 2019, Finnish Institute for Health and Welfare: Helsinki. p. 132.

47. Finell, E., Tolvanen, A., Pekkanen, J., Minkkinen, J., Stahl, T., and Rimpela, A., *Psychosocial Problems, Indoor Air-Related Symptoms, and Perceived Indoor Air Quality among Students in Schools without Indoor Air Problems: A Longitudinal Study.* Int J Environ Res Public Health, 2018. **15**(7).

48. Palmquist, E., Stenberg, B., Neely, G., and Nordin, S., *Environmental intolerance and mental ill-health: which comes first?* 2017.

**List of Supplemental Digital Content**Supplemental Digital Content 1.pdf

**Figure legend**

Figure 1. Prevalence of functional impairment (i.e. difficulties with work, managing domestic responsibilities, or social life) due to indoor air-related symptoms in the past 12 months

Figure 2. Proportion of self-reported symptoms (at least once or twice a week) by different levels of functional impairment
